# Assessing the Physical Activity Questionnaire for Adolescents (PAQ–A): Specific and General Insights from an Ethiopian Context

**DOI:** 10.1155/2021/5511728

**Published:** 2021-07-14

**Authors:** Eshetu Andarge, Robert Trevethan, Teshale Fikadu

**Affiliations:** ^1^School of Public Health, Arba Minch University, PO Box 021, Arba Minch, Ethiopia; ^2^Albury, NSW, Australia

## Abstract

The Physical Activity Questionnaire for Adolescents (PAQ–A) has been used in a variety of forms and in a range of countries. This study involves a detailed examination of the PAQ–A to determine its applicability and effectiveness in an Ethiopian setting. We administered the scale to 110 Ethiopian adolescents on two occasions, 5 weeks apart. Data were inspected for features typical of the participants and analyzed to identify interitem correlations, the scale's factor structure, and a range of descriptive statistics concerning composite scores. Most of the scale's items were satisfactorily interrelated according to lenient criteria, and most items loaded on a single factor in exploratory factor analyses. However, a number of the scale's properties were deficient according to stringent or conventionally accepted psychometric criteria. Close inspection of participants' responses highlighted problems in the way the scale is worded, interpreted by participants, and scored. Although the scale does not capture PA as an homogeneous construct, we argue that this is not a problem and neither is its poor test–retest reliability. We make recommendations concerning presentation and scoring of the PAQ–A that are likely to enhance its validity beyond Ethiopia, and we provide a modified version of the scale.

## 1. Introduction

Adolescence is a time during which physical activity (PA) is susceptible to influence [1, 2] and when lifelong habits are established concerning PA. These habits have consequences for subsequent physical and mental health [3–6]. It is therefore concerning that global statistics in 2016 indicated that 81.0% of students aged 11–17 years had insufficient levels of PA, with that figure rising to 84.9% in low-income countries [7]. Under these circumstances, it is important to measure PA accurately in order to identify the extent of physical inactivity in particular geographic areas; to characterize adolescents' PA appropriately, particularly if intergroup comparisons are of interest; to monitor trends in PA; and to evaluate the effectiveness of interventions. In this article, we explore whether the Physical Activity Questionnaire for Adolescents can provide the foundation for meeting these aims.

### 1.1. The Physical Activity Questionnaire for Adolescents

For the last two decades, one way in which physical activity has been assessed among adolescents has been the Physical Activity Questionnaire for Adolescents (PAQ–A) [8]. This self-report scale, described by its creators as having been designed for use with school students in grades 9 to 12, contains eight items intended to capture adolescents' recollection of their PA over the preceding 7 days. The first and last of the PAQ–A's eight items each contains a number of subitems from which a mean is initially calculated, and those two means are added to responses on the other six items to obtain a total from which the mean is calculated to produce a composite score ranging from 1 to 5, with higher scores indicating greater PA. A ninth question seeks information about anything that would have prevented respondents from engaging in their “normal physical activities” during the previous week.

The PAQ–A is free of copyright constraints, inexpensive to produce, easy and quick to administer, and easy to respond to and score. It is also modifiable according to contexts in which it is administered, which could account for research with the PAQ–A having been conducted in a range of countries, including Brazil [9], Canada [10–13], Ghana [14], Nigeria [15, 16], Poland [17], Spain [18, 19], the Netherlands [20], the UK [21, 22], and the USA [23, 24].

### 1.2. Psychometric Attributes of the PAQ–A

Nearly a decade ago, the PAQ–A attracted strong approbation for its psychometric attributes when Biddle et al. [25] identified 89 scales intended to measure physical activity in young people. After reducing their selection to 20 instruments, primarily based on the requirement that reliability and/or validity had been demonstrated, the researchers and most members of an expert panel endorsed only the PAQ–A (in conjunction with its companion version for children, the PAQ–C) and two other PA scales.

Congruent with this endorsement, Wyszyńska et al. [17] recently obtained impressive psychometric results when administering the PAQ–A to a sample of 78 Polish school students. After data of two students who indicated reduced PA in the previous week had been removed, the lowest item–total correlation was 0.43 and 75% of the item–total correlations exceeded 0.50, coefficient alpha was 0.93, the intraclass correlation coefficient (ICC) associated with test–retest reliability was 0.97, and high correlations (0.81 and 0.94) were obtained with accelerometer results.

From a small number of publications that contain more than basic information about the PAQ–A's psychometric properties, it is possible to identify several features of the PAQ–A that are noteworthy. When item–total or corrected item–total, correlations have been reported; the lowest of these correlations is often considerably lower than the lowest value of 0.43 obtained by Wyszyńska et al. [17]. In other research, these correlations lie between 0.23 and 0.70, among which the correlations associated with Item 3 (concerning lunch-time PA) have been identified as the lowest [11, 21]. Item 3 was also rated as the least relevant in an item-level content validity index analysis [20], and the same item carried noticeably weak correlations with accelerometry-derived PA and with results on two other self-report PA scales [21]. Item 3, therefore, could be regarded as problematic.

There appears to be limited interest in or assessment of the PAQ–A's factor structure. In the only relevant study that we located, Janz et al. [23] conducted several exploratory factor analyses and concluded that there was a single factor in the scale. However, their analyses could be compromised because data were initially obtained from the participants at age 11 and were supplemented with data from the same participants 2 years later to provide the full data set. Furthermore, no indication was provided about the strength of communalities or percentage of variance accounted for in any of the analyses. Item 3 had the lowest factor loadings (from 0.12 to 0.18), although activity associated with PA classes (Item 2) also had weak loadings in some analyses.

Among the reported coefficient alphas, the value of 0.93 obtained by Wyszyńska et al. [17] is atypically high, with other researchers reporting alphas ranging from 0.65 to 0.84 [9, 11, 19–21, 23].

Concurrent validity has been assessed in a number of studies. In several of these, PAQ–A scores were correlated with accelerometer readings and yielded only moderate correlations of 0.33 [10], 0.34 and 0.39 [19], 0.35 [24], 0.39 and 0.42 [21], and 0.44 to 0.55 [11]. Janz et al. [23] found stronger correlations of 0.56 and 0.63 between the PAQ–A and accelerometer readings after having made a number of alterations to the PAQ–A, and Kawalski et al. [10] correlated PAQ–A scores with scores on four other self-report PA scales and obtained correlations ranging from 0.51 to 0.73, revealing that self-reports yield higher correlations with each other than does the PAQ–A with physiological indicators of PA [10]. Together, these results provide only moderate evidence of concurrent validity.

Convergent validity was assessed in two ways by Aggio et al. [21], who asked adolescents to indicate their self-perceptions based on the International Fitness Scale and the Physical Activity Self-Efficacy Scale. The correlations with PAQ–A scores were 0.35 and 0.32, respectively. Bervoets et al. [20] correlated PAQ–A scores with results on a cardiopulmonary exercise test and obtained a correlation of 0.52. As with concurrent validity, these results are not reassuring.

In addition to the study by Wyszyńska and her colleagues [17], test–retest reliability has been assessed in a number of studies [9, 11, 19, 23], but the results are difficult to place confidence in or to summarize because unconventional practices have sometimes been used [23]; disparate amounts of time have occurred between administrations ranging from 1 week to 2 years [9, 11, 17, 19, 21, 23]; different statistics have been used [9, 11, 19, 21, 23]; a wide variety of findings have been reported, with Spearman's correlations ranging from 0.30 to 0.39 [23] and ICCs ranging from 0.68 to 0.91 [9, 11, 17, 19, 21]; information concerning the model, form, and type of the ICC used in the analyses is almost never provided despite these aspects sometimes influencing ICC values substantially, and researchers have used too-lenient criteria for describing their ICC results [11, 26, 27].

In summary, there are a number of indications that neither the PAQ–A itself, nor the way in which it has been assessed, are highly satisfactory. This is reflected in two review studies in which the outcomes differ from those in the review by Biddle et al. [25]. For example, after examining 61 PA scales, including the PAQ–A, Chinapaw et al. concluded that no PA scale demonstrated acceptable reliability and validity [28]. In a follow-up systematic review, Hidding et al. [29] examined 162 studies in which 89 PA scales (again including the PAQ–A) had been evaluated. The researchers concluded that evidence of acceptable reliability and validity was available for none of the 89 scales—although ironically that was usually because of poor methodological quality exhibited by the studies in which the scales were evaluated, not because of deficiencies in the scales themselves.

Two conclusions are evident from the above. One is that the PAQ–A's properties, although perhaps better than those of other PA scales, are not consistently impressive. The other is that the scale's properties have not been adequately assessed. There is therefore a need to draw a distinction between the quality of the PAQ–A and the quality of the studies in which it has been examined. Both of these issues are pursued within the present research in which we explored the appropriateness and psychometric properties of the PAQ–A in an Ethiopian setting. In doing so, we were responding to the comments by Hidding et al. that “high-quality methodological studies examining all relevant measurement properties are highly warranted” [29] and by Biddle et al. that, for scales such as the PAQ–A, researchers should “evaluate their appropriateness for application within their national context” [25]. In this case, the national context is Ethiopia, but we believe a number of outcomes from our research have wider applicability.

## 2. Methods

### 2.1. Participants

A total of 120 adolescents were selected for this research, all from Southern Ethiopia. They comprised 40 students from each of two high schools in Arba Minch and 40 students from one high school in Jinka. In each school, 20 boys and 20 girls were selected such that there would be 10 students from each of grades 9 to 12. Among those selected to participate, none had a physical disability or visual impairment.

Ethics approval for the study was obtained from the Institutional Ethics Review Board of the College of Medicine and Health Sciences, Arba Minch University, Ethiopia—approval number IRB/116/11, dated 02/09/2019. Letters of permission were initially obtained from the education offices at Arba Minch and Jinka, and permission to conduct the study was subsequently obtained from all three schools. Rather than asking students to provide prior signed consent, students were told that participation in the research was not compulsory and that they could choose to opt out at any point without negative consequences—a strategy that was likely to offer them greater freedom concerning whether they wanted to participate.

### 2.2. The PAQ–A

Thirteen of the 22 types of PA listed within Item 1 of the PAQ–A were removed as irrelevant or uncommon in an Ethiopian context, and no types of PA were added to the activities within that item. The scale was translated from English to Amharic by English and Amharic language teachers and a physical education teacher. Back translation was not employed in light of the lack of linguistic and conceptual complexity within the scale.

### 2.3. Procedure

Questionnaires were administered in hard copy form immediately after the final class for the day. For test–retest purposes, students were asked to participate on two occasions, with a 5-week gap between administrations, and on both occasions, students were reassured that participation in the research was not compulsory. Questionnaires were administered in hard copy form under supervision of the researchers who attempted to ensure that no data were missing.

At Time 1, in addition to responding to the PAQ–A, participants were asked to indicate their sex and age. They were also asked to provide their student number to enable matching with the retest data. At Time 2, participants were asked to provide only responses to the modified PAQ–A and their student number.

## 3. Results

### 3.1. Participants

At Time 1, three students indicated that they were unwilling to participate in the research, one male student completed the questionnaire within 2 minutes and chose the first option for all items, and six female students did not respond to most items. The data for all 10 of these students were excluded, bringing the number of participants retained for analyses at Time 1 to 110. Among these 110 students, there were 57 boys and 53 girls. Their ages ranged from 14 to 19 (*M* = 16.35, SD = 1.38). Ten of them indicated that circumstances over the previous week had reduced the amount of PA they would normally engage in. These circumstances included injury, illness, and menstruation.

At Time 2, data were sought from only the 110 students whose responses had been retained from the Time 1 administration, and no students opted out of participation. One male student chose the first option for all items, but his data were retained because he had provided more varied responses at Time 1. Therefore, data from all 110 respondents were retained for analysis at Time 2. Among these participants, 17 indicated that circumstances over the previous week had reduced the amount of PA they would normally engage in. These circumstances were predominantly injuries and illness. None of these 17 participants were among the 10 who had reported PA-limiting circumstances at Time 1.

### 3.2. Initial Inspection of Data

Responses to the listed activities in Item 1 indicated high involvement by five participants on almost all of those activities at Time 1 and by two participants at Time 2. Although that pattern of responding suggested misunderstanding, carelessness, or attempts to sabotage the research, we did not alter their data in any way because we had not seen mention of data cleaning in other studies in which the PAQ–A has been used and because we anticipated the effect of an inflated score on Item 1 would be minimized when the composite score was calculated. On both occasions of measurement, the most commonly chosen option within Item 1 was walking for exercise, followed by jogging/running, aerobics, dance, and football.

Across all items on both occasions, there was a strong tendency for the first three response options, indicating lower levels of PA, to be chosen. According to Item 8, PA was evenly spread across all days of the week on both occasions of measurement, with a slight increase on Saturday.

For both times of administration, means and SDs on all items, and the eight-item composite score, are shown in Table 1. The highest mean score at Time 1 was 2.39 on Item 2, relating to physical education (PE) classes. However, 24.5% of the participants indicated that they had not attended a PE class in the previous week, and of the remainder, 84.3% indicated they were hardly ever or only sometimes physically active in those classes. At Time 2, the highest mean on an individual item was 2.52, again on Item 2 relating to PE classes, and among the participants who were doing PE, 77.3% were hardly ever or only sometimes physically active in those classes. Item 8 (averaged frequency of PA across the week) was the next highest at Time 1 and the equal second highest with Item 5 (evening activity) at Time 2. Relative to the other items, Item 1 had a narrow SD on both occasions of measurement.

Because of our concerns about Item 8 being susceptible to poor recall, we inspected responses on it closely and found that participants' scores on that item, despite being a combination of separate PA frequencies across the previous seven days that might therefore have provided a relatively stable overview of PA, differed by ≥1 between the two occasions of measurement for 35 (32%) of the participants. In contrast, Item 1, which is also based on a number of subitems, had only 23 (21%) of the participants with scores that differed by ≥1 between the two occasions of measurement. Scores on Item 8 seemed, therefore, to be particularly inconsistent across time.

For both measurement occasions, the highest interitem correlations were between after-school PA and evening PA (0.60 and 0.43 at Times 1 and 2, respectively), and evening PA had the highest corrected item–total correlations (0.61 and 0.55 at Times 1 and 2, respectively).

At Time 1, 22 (78.6%) of the 28 interitem correlations were between 0.15 and 0.50, with only one ≥ 0.50 (at 0.60). The mean of these correlations was 0.26. Of six interitem correlations below 0.15, four were associated with Item 3 (lunch-time activity). The eight corrected item–total correlations ranged from 0.22 to 0.61, with Item 3 having the only correlation ≤ 0.30.

The KMO index was 0.75, and for Bartlett's test of sphericity, *p* < 001—thus indicating data factorability. Parallel analysis indicated the presence of only one factor in the data. An initial exploratory factor analysis with principal axis factoring as the method of extraction and constrained to a single factor revealed the eight items accounting for only 35.71% of the variance. The extraction communalities were generally low (mean of 0.28), with Items 3 and 7 (the latter referring to general free-time activity) having extraction communalities exceptionally low at < 0.15. Both of those items also had factor loadings < 0.40. Because of the poor performance of Item 3 on several criteria, we conducted a second exploratory factor analysis after deleting it. This resulted in 39.8% of the variance being accounted for, and although the communality for Item 7 was still unacceptably low (at 0.15), it loaded at 0.38. The other seven items had loadings that ranged from 0.46 to 0.72. Refer to Table 2.

Despite some of the above results being unimpressive, we decided neither to eliminate Item 7 in a subsequent exploratory factor analysis nor to explore the possibility of eliminating any other items in factor analyses subsequent to that, until we had analyzed the data from Time 2.

At Time 2, 22 (78.6%) of the 28 interitem correlations were between 0.15 and 0.50, with none ≥ 0.50. The mean of these correlations was 0.26, and 16 (57.1%) were ≤ 0.30. Of six correlations below 0.15, five were associated with Item 3. The eight corrected item–total correlations ranged from 0.19 to 0.55, with only one of these correlations ≤ 0.30, again for Item 3.

At Time 2, the KMO index was 0.79, and for Bartlett's test of sphericity, *p* < 001. Parallel analysis again clearly indicated that there was only one factor in the data. An initial exploratory factor analysis constrained to a single factor revealed the eight items accounting for only 36.59% of the variance. Extraction communalities were generally low (mean = 0.28), with Items 1 and 3 exceptionally low at < 0.18, but only Item 3 had a factor loading < 0.40. The other items loaded between 0.42 and 0.66. Because of the poor performance of Item 3 on several criteria, we conducted a second exploratory factor analysis after deleting that item. This resulted in 40.96% of the variance being accounted for, and although the communality for Item 1 was still unacceptably low (at 0.15), that item had a loading of 0.39 which we regarded as minimally acceptable. The other seven items had loadings that ranged from 0.46 to 0.66. Refer to Table 2.

Because of its poor performance on both measurement occasions, we discarded Item 3 in all subsequent analyses, thus creating a modified version of the scale. Because the psychometric attributes of Item 1 were similar to the attributes of the other items at Time 1 despite being poorer at Time 2, but the reverse applied for Item 7, we believed that both of those items could justifiably be retained.

For both occasions of measurement, composite PA scores were calculated for each participant according to the standard procedures for the PAQ–A, with the exception that Item 3 was omitted. When outliers were defined as composite scores within the first or fourth quartiles that exceeded 1.5x the interquartile range, there were three high outliers at Time 1 and one high outlier at Time 2. None of the participants had outliers on both occasions. For three of the four participants with outliers, responses on Item 8 were noticeably higher than their responses on all other items. However, including or excluding this item made little difference when these participants' composite scores were computed, so we made no adjustment because of it.

Table 3 contains results on the modified PAQ–A scale data at both occasions for the whole sample as well as for a smaller, more refined, subsample (*n* = 79) in which participants were excluded if they had reduced activity or outlying data on either measurement occasion.

Test–retest reliability was assessed with paired-samples *t*-tests, Pearson's correlation coefficients, and ICCs (3, 1 (two-way mixed effects, single measures) absolute agreement). For each of these metrics, two sets of analyses were conducted on each measurement occasion, one set based on the total sample and the other on the smaller, more refined subsamples comprising participants who had neither reduced PA nor outlying data on either measurement occasion. We adopted this strategy in case temporary reductions in PA for some participants influenced the results and also because of the recommendation that analyses should occur both with and without outliers [30]—and particularly in this case, because outliers can distort Pearson's correlations and ICCs [31].

Results from the two sets of analyses are summarized in Table 4. Both *t*-tests were nonsignificant, indicating neither upward nor downward movement in scores across the 5-week period, as is strongly suggested by the similarity of the composite score means in Table 3. In contrast, at 0.33 and 0.43, neither of the Pearson's correlations could be regarded as impressive for test–retest purposes despite being statistically significant. Furthermore, both ICCs fall well below 0.50 and therefore can be regarded as poor.

## 4. Discussion

### 4.1. Discussion Concerning Results in This Study

This study provides insights concerning use of the PAQ–A, not only among Ethiopian adolescents but more broadly. Of importance, our having found that a single factor in the data is congruent with results from the study by Janz et al. [23], and both sets of results support an assumption that appears to run through the research involving the PAQ–A that there is no need to create subdomains within the scale. However, despite the interitem and corrected item–total correlations being acceptable [32, 33] and therefore suggesting a sufficiently unified focus for the scale, the generally low extraction communalities, the consistently low percentages of variance accounted for in the data (never exceeding 41%, even after Item 3 had been removed), and the moderate coefficient alphas all indicate a lack of cohesion among the items. This raises the possibility that items on a PA scale need not be highly correlated [28] and that PAQ–A scores comprise a desirable variety of components that can differ from one adolescent to another in relation to the nature of PA, the time when PA occurs, the duration of PA, and the extent of exertion. In light of that, our having removed Item 3 from the final analyses because of its low association with other items was probably inappropriate and even counterproductive. Furthermore, the common desire among researchers for their data to exhibit high coefficient alpha values could be regarded as misguided in relation to the PAQ–A.

Given that participants used all five options on all items at Time 1 and on all but two items at Time 2, the results in the present study indicate that the range of response options on the PAQ–A is appropriate. However, the narrow SDs in this research and in other research [9, 10, 15, 17–21, 23, 24] suggest that the scale is incapable of differentiating adolescents unless their differences in PA are large. That might not be a defect in the scale, however, because the scores congregated at the lower end of the distribution and PA is widely known to be low among adolescents [7]. The PAQ–A might therefore represent adolescents' PA accurately. In that respect, Item 7, with its narrow SDs and the lowest mean on both occasions of measurement, could be one of the most salient items despite not performing well in the factor analyses.

In the test–retest analyses, the nonsignificant paired-samples *t*-tests indicate that the PAQ–A is not so inherently unstable that scores on it change in the absence of pervasive influences [27, 34] such as ambient temperature and inclement weather—or, particularly for school students, influences such as studying for examinations, concentrated involvement with extracurricular activities, or being on vacation. Our results also suggest, reassuringly, that initial exposure to the PAQ–A does not influence responses at a subsequent point in time, for example, by encouraging adolescents to engage in more PA.

The low test–retest ICCs in our research, and ICCs being< 0.75 in other research [9, 11, 19], suggest that many adolescents' PA is inconsistent from one time to another. This is reinforced by the improvement in ICCs being only slight when 31 students were removed from the dataset to produce the more refined subsample. It follows that if adolescents habitually and validly have inconsistent PA, even over brief spans of time, the raison d'être for assessing test–retest reliability for PA scales dissolves, and rather than assessing PA at different timepoints to assess temporal consistency, energy might be better directed at obtaining samples of adolescents' PA at two or more timepoints and averaging the results, to characterize adolescents' PA more accurately than is possible by assessing their PA at only one timepoint.

When composite scores were obtained after Item 3 was discarded, unanticipated psychometric insights emerged following removal of participants with reduced activity or outliers from the analyses. The first was that departures from a normal distribution might be resolved. Although the Shapiro–Wilk test indicated a significant departure from normality in the total sample's data at Time 1, this lack of normality was not present in data from the refined subsample at that occasion of measurement. Furthermore, although the means and SDs differed little between the total and refined samples, the test–retest Pearson's correlations and ICCs were noticeably higher and therefore more desirable, with data from the refined samples. These outcomes indicate that different results as well as psychometric improvements can result from removing records with atypical data.

### 4.2. Suggested Improvements to the PAQ–A

Despite satisfactory or even admirable features of the PAQ–A, our close inspection of the data suggests that some aspects of the scale are unsatisfactory either with its inherent characteristics or with the way in which it can be easily misperceived and inappropriately responded to by participants. Because walking for exercise was the most common form of PA identified among the choices in Item 1, the participants appear not to have appreciated that they were being asked about brisk walking. Given typical habits of Ethiopian adolescents, they appear to have responded as if they were being asked about walking from one location to another in the normal course of events, possibly including strolling casually with friends.

It might therefore be necessary for those administering the scale, regardless of context, to emphasize a need for participants to focus on prolonged exertion to the point of breathing “hard.” Although that focus is indicated in the scale's initial instructions, greater emphasis might be given in the wording of some items. For example, the Item 1 subitem about walking could be altered to something such as “Walking quickly for exercise.” Placing this subitem after other subitems such as bicycling, jogging, and aerobics might also be advantageous by indicating that strenuous, not casual, walking is the target. We believe that italicizing important words in the instructions for all items would also be beneficial.

These suggestions are implemented in the version of the PAQ–A that we provide in the Supplementary Material (available here) of this article. That version includes Item 3 according to our decision that it serves a useful purpose despite often not carrying a high association with the other items.

While examining Item 8 (relating to how often participants had been engaged in physical activity on each day during the previous week), we realized that the first three response options of *none*, *a little bit*, and *medium* are semantically discordant relative to that item's question (which focuses on frequency). Furthermore, the five response options for this item are discordant with each other because the first two refer to extent and the final two to frequency, and the middle option (*medium*) is confusing because it is not clear whether that option refers to a medium amount in terms of extent, frequency, duration, other adolescents' PA, or perhaps something else. This kind of ambiguity is likely to lead to measurement error [35]. We therefore suggest that the duration be the consistent response-option target of Item 8 not only to avoid the current incompatible set of options but also because duration of PA is tapped by none of the other items. An array of response options such as *no time*, *about 15 minutes*, *about 30 minutes*, *about 45 minutes*, and *an hour or more* is likely to be appropriate. Alterations to this item, reflecting this suggestion, are also indicated in the Supplementary Material (available here).

Scoring of the PAQ–A might also be improved. When we examined responses to individual activities in Item 1, we realized that the way scores on that item are calculated can easily distort participants' PA scores downward. For example, if participants jog each day for an hour and that is their only form of PA among the 22 activities listed on Item 1, they would attract a score of only 1.18 on that item despite satisfying the World Health Organization recommendation of ≥60 minutes of daily moderate to vigorous physical activity [36]. Scores on Item 1 are highly likely to be inaccurately low in a variety of other ways, all of which result in a cluster of scores at the lower end of the distribution for that item. One possibility for overcoming this problem would be for participants who respond with the first option on all Item 1 activities to be given a score of 1 on that item, and that, for participants who indicate involvement in only some of the activities, their responses on those activities be used for determining the nominator on this item, with the denominator being the number of activities that had responses greater than 1. According to the above example, participants who jogged for at least an hour a day and were engaged in none of the other activities would be accorded a score of 5—thus indicating, accurately, a high level of PA for that item. However, we harbor sufficient doubts about the validity of this calculation to recommend that researchers use Item 1 only to identify the predominant kinds of PA that specific adolescents, or groups of adolescents, are engaged in (an aspect of PA that is interesting in its own right) and not incorporate it when calculating a composite PA score.

Whether the above alterations would result in more valid PA scores is a matter for future research, but it is tempting to speculate about the extent to which previous research outcomes would have been different and more impressive if those alterations had previously been in place. This includes the possibility of less mediocre results when assessing construct validation for the PAQ–A by correlating scores on it with accelerometer data [10, 19, 21, 23, 24].

### 4.3. Strengths and Limitations of This Research

Strengths of this research include the participants spanning the school grades for which the PAQ–A was designed, there being similar numbers of boys and girls, and the high response and retention rates. In addition, the test–retest interval was not so brief that similar PA across the timespan might have created an inaccurately high impression of temporal stability in PA. We have also provided a thorough examination of the psychometric properties of the PAQ–A. The main limitations are generalizability of the results because the samples were drawn from urban areas within only one region of Ethiopia, and that apart from modifying the activities listed in Item 1, we conformed with how most other researchers appear to have used the PAQ–A rather than altering the scale in order to effect and report what we believe would have been improvements to its presentation and scoring.

## 5. Conclusions

Despite the developers of the PAQ–A acknowledging it is difficult to assess intensity, frequency, and duration of PA, the scale appears capable of identifying, from a variety of facets and timepoints during a week, the kind of moderate to vigorous PA that the World Health Organization recommends adolescents be involved in for ≥60 minutes a day. This is largely because Items 2 to 7 tap intensity and frequency of PA if those items are responded to appropriately, and Item 8 can be improved with little adjustment. The scale has an added advantage of being flexible in that researchers can adjust the first item to suit local conditions, and its brevity is unlikely to attract satisficing by participants. The scale also permits identification of specific aspects of adolescent PA.

This research provides considerable support for use of the PAQ–A, but we believe the scale could be improved for use in both Ethiopian and wider contexts by its instructions emphasizing a focus on vigorous physical activity, rewording some items and response options, and not using Item 1 as part of the overall score but solely to obtain an indication of the kinds of PA that participants are engaged in. We also believe that researchers should analyze PAQ–A data with total samples as well as with refined samples in which records from participants with reduced activity and outliers have been removed—and report results from both sets of analyses.

## Figures and Tables

**Table 1:** Means and SDs of PAQ–A items and composite score on each occasion of measurement.

Item	Time 1		Time 2	
	Mean	SD	Mean	SD
(1) Spare time activities	2.01	0.69	1.88	0.54
(2) In PE classes	2.39	1.04	2.52	1.07
(3) Lunch time	1.86	0.96	1.93	1.08
(4) After school	2.30	1.15	2.25	1.06
(5) Evening	2.28	1.17	2.33	1.02
(6) Weekend	2.16	1.00	2.25	0.92
(7) Exertion, free time	1.83	0.95	1.80	0.78
(8) Frequency, each day	2.32	0.92	2.30	0.81
Composite score^a^	2.15	0.59	2.15	0.54

^a^The composite score is based on all eight items.

**Table 2:** Extraction communalities and factor loadings of retained PAQ–A items at each occasion of measurement^a^.

Item	Time 1		Time 2	
	Communalities	Loadings	Communalities	Loadings
(1) Spare time activities	0.27	0.52	0.15	0.39
(2) In PE classes	0.26	0.51	0.38	0.62
(4) After school	0.40	0.64	0.33	0.58
(5) Evening	0.51	0.72	0.43	0.66
(6) Weekend	0.21	0.46	0.27	0.52
(7) Exertion, free time	0.15	0.38	0.22	0.46
(8) Frequency, each day	0.31	0.56	0.42	0.65

^a^Results in this table are based on seven items (excluding Item 3 relating to lunch-time PA).

**Table 3:** Characteristics of composite scores on the modified PAQ–A at each occasion of measurement^a^.

Metric	Time 1		Time 2	
	Total sample	Refined subsample^b^	Total sample	Refined subsample
Coefficient alpha	0.74	0.69	0.76	0.73
Physical activity				
Minimum/maximum	1.00/3.99	1.00/3.46	1.00/4.13	1.00/3.23
Mean	2.19	2.14	2.20	2.16
SD	0.63	0.57	0.57	0.54
Median	2.16	2.13	2.20	2.19
Interquartile range	0.76	0.68	0.71	0.76
Skewness (*z*)	2.04	0.87	0.80	−0.52
Kurtosis (*z*)	0.25	−0.24	0.63	−0.98
Normality^c^	0.97, 110, 0.029	0.98, 79, 0.200	0.99, 110, 0.417	0.98, 79, 0.315

^a^Results in this table are based on seven items (excluding Item 3 relating to lunch-time PA). ^b^The refined subsample (*n* = 79) comprised participants from the total sample (*N* = 110) who had neither a reduced amount of physical activity nor outlying physical activity scores on either occasion of measurement. ^c^Normality results are presented as the Shapiro–Wilk *W* value, df, and *p* value.

**Table 4:** Results related to test-retest analyses^a^.

Metric	Total sample (*N* = 110)	Refined subsample (*n* = 79)^b^
Paired-samples *t*-test	*t*(109) = 0.06, *p* = 0.951	*t*(78) = 0.42, *p* = 0.675
Pearson's product moment correlation	0.33, *p* < 0.001	0.43, *p* < 0.001
Intraclass correlation coefficient^c^	0.34 (0.16, 0.49)	0.43 (0.24, 0.60)

^a^Results in this table are based on seven items (excluding Item 3 relating to lunch-time PA). ^b^The refined subsample (*n* = 79) comprised participants from the total sample (*N* = 110) who had neither a reduced amount of physical activity nor outlying physical activity scores on either occasion of measurement. ^c^The 95% confidence interval is shown in brackets.

## Data Availability

The datasets used and/or analyzed during the current study are available from the corresponding author on reasonable request.

## Abbreviations

ICC: Intraclass correlation coefficient
MVPA: Moderate-to-vigorous physical activity
PA: Physical activity
PAQ–A: Physical Activity Questionnaire for Adolescents
WHO: World Health Organization.

## Supplementary Materials

Supplementary Materials The modified questionnaire of the PAQ–A.
