# Supplementary material for: Assessing the Physical Activity Questionnaire for Adolescents (PAQ–A): Specific and General Insights from an Ethiopian Context
Source: Biomed Res Int. 2021 Jul 14;2021:5511728. doi: 10.1155/2021/5511728 (PMC8294967; doi:10.1155/2021/5511728)
Supplement: Supplementary Materials — The modified questionnaire of the PAQ–A. [file 5511728.f1.docx]

**Modified Version of the PAQ–A**

We are trying to find out about your level of physical activity in *the last 7 days*.

We want to focus on any activities, including games, that make you move quickly and breathe hard.

There are no right or wrong answers. This is not a test. Please answer all questions as honestly and accurately as you can.

1. In your spare time during the *past 7 days* (the last week), did you get exercise from any of the following activities? Circle a number for each activity.

|  |  | No | 1–2  times | 3–4  times | 5–6  times | 7 or more times |
| --- | --- | --- | --- | --- | --- | --- |
| a | Bicycling | 1 | 2 | 3 | 4 | 5 |
| b | Jogging or running | 1 | 2 | 3 | 4 | 5 |
| c | Aerobics | 1 | 2 | 3 | 4 | 5 |
| d | Walking quickly for exercise | 1 | 2 | 3 | 4 | 5 |
| e | Swimming | 1 | 2 | 3 | 4 | 5 |
| f | Dancing | 1 | 2 | 3 | 4 | 5 |
| g | Football | 1 | 2 | 3 | 4 | 5 |
| h | Volleyball | 1 | 2 | 3 | 4 | 5 |
| i | Basketball | 1 | 2 | 3 | 4 | 5 |
| j | Other kinds of vigorous exercise | 1 | 2 | 3 | 4 | 5 |

What other kinds of vigorous exercise did

you do in addition to things in the list above? ______________________________________

______________________________________

2. In the last *7 days*, during your physical education (PE) classes, how often were you *very active*
(e.g., playing hard, running, jumping, or throwing)? Circle only one number.

I don’t do PE 1

Hardly ever 2

Sometimes 3

Quite often 4

All of the time 5

3. In the last 7 days, what did you normally do at *lunch time* (besides eating lunch)? Circle only one number.

Sat down (talked, read, did schoolwork) 1

Stood around or walked around 2

Was physically active for some of the time 3

Was physically active for quite a lot of the time 4

Was physically active for most of the time 5

4. In the last 7 days, on how many days *right after school* were you *very active* in sport, playing games, dancing, swimming, etc.? Circle only one number.

None 1

1 time last week 2

2 or 3 times last week 3

4 times last week 4

5 times last week 5

5. In the last *7 days*, on how many *evenings* were you *very active* in sport, playing games, dancing, etc.? Circle only one number.

None 1

1 time last week 2

2 or 3 times last week 3

4 times last week 4

5 times last week 5

6. On the *last weekend*, how many times were you *very active* in sport, playing games, dancing, etc.? Circle only one number.

None 1

1 time 2

2 – 3 times 3

4 – 5 times 4

6 or more times 5

7. Which of the following statements describes you best for the last *7 days*? Circle only one number.

All of most of my free time was spent doing things that involve little physical effort 1

I sometimes (1 or 2 times last week) was physically very active in my free time (e.g., did sport, jogged, did aerobics, swam, rode a bike) 2

I often (3 or 4 times last week) was physically very active in my free time (e.g., did sport, jogged, did aerobics, swam, rode a bike) 3

I quite often (5 or 6 times in the last week) was physically very active in my free time (e.g., did sport, jogged, did aerobics, swam, rode a bike) 4

I very often (7 or more times last week) was physically active in my free time 5

8. For each day last week, please estimate approximately the *total* amount of time you were *very active* doing such things as playing games, doing sport, jogging, or dancing.
Circle one number for each day.

|  |  | No time | About 15 minutes | About 30 minutes | About 45 minutes | An hour  or more |
| --- | --- | --- | --- | --- | --- | --- |
| 1 | Monday | 1 | 2 | 3 | 4 | 5 |
| 2 | Tuesday | 1 | 2 | 3 | 4 | 5 |
| 3 | Wednesday | 1 | 2 | 3 | 4 | 5 |
| 4 | Thursday | 1 | 2 | 3 | 4 | 5 |
| 5 | Friday | 1 | 2 | 3 | 4 | 5 |
| 6 | Saturday | 1 | 2 | 3 | 4 | 5 |
| 7 | Sunday | 1 | 2 | 3 | 4 | 5 |

Were you sick last week, or did anything prevent you from doing your normal physical activities?

Yes 1 Why did you answer yes? __________________________________

No 2
